# Global end-diastolic volume acquired by transpulmonary thermodilution depends on age and gender in awake and spontaneously breathing patients

**DOI:** 10.1186/cc8209

**Published:** 2009-12-14

**Authors:** Stefan Wolf, Alexander Rieß, Julia F Landscheidt, Christianto B Lumenta, Patrick Friederich, Ludwig Schürer

**Affiliations:** 1 Department of Neurosurgery, Klinikum Bogenhausen, Akademisches Lehrkrankenhaus der Technischen Universität München, Englschalkinger Straée 77, München 81925, Germany; 2 Department of Anesthesiology, Klinikum Bogenhausen, Akademisches Lehrkrankenhaus der Technischen Universität München, Englschalkinger Straée 77, München 81925, Germany; 3 Department of Neurosurgery, Charité Campus Virchow, Freie Universität Berlin, Augustenburger Platz 1, Berlin 13353, Germany

## Abstract

**Introduction:**

Volumetric parameters acquired by transpulmonary thermodilution had been repeatedly proven superior to filling pressures for estimation of cardiac preload. Up to now, the proposed normal ranges were never studied in detail. We investigated the relationship of the global end-diastolic volume (GEDV) acquired by transpulmonary thermodilution with age and gender in awake and spontaneously breathing patients.

**Methods:**

Patients requiring brain tumor surgery were equipped prospectively with a transpulmonary thermodilution device. On postoperative day one, thermodilution measurements were performed in 101 patients ready for discharge from the ICU. All subjects were awake, spontaneously breathing, hemodynamically stable and free of catecholamines.

**Results:**

Main finding was a dependence of GEDV on age and gender, height and weight of the patient. Age was a highly significant non-linear coefficient for GEDV with large inter-individual variance (p < 0.001). On average, GEDV was 131.1 ml higher in males (p = 0.027). Each cm body height accounted for 13.0 ml additional GEDV (p < 0.001). GEDV increased by 2.90 ml per kg actual body weight (p = 0.043). Each cofactor, including height and weight, remained significant after indexing GEDV to body surface area using predicted body weight.

**Conclusions:**

The volumetric parameter GEDV shows a large inter-individual variance and is dependent on age and gender. These dependencies persist after indexing GEDV to body surface area calculated with predicted body weight. Targeting resuscitation using fixed ranges of preload volumes acquired by transpulmonary thermodilution without concern to an individual patient's age and gender seems not to be appropriate.

## Introduction

Therapy of severe circulatory dysfunction is dependent on a reliable estimation of cardiac preload. Transpulmonary thermodilution offers accurate measurement of cardiac output (CO) and the assessment of preload filling volumes. In comparison with central venous pressure and pulmonary capillary wedge pressure, estimation of preload using transpulmonary thermodilution derived global end-diastolic volume (GEDV) or intrathoracic blood volume (ITBV) has been repeatedly proven to be superior [1-5]. Consistently, filling pressures are considered inadequate for guiding volume therapy [6].

GEDV is a hypothetical volume that assumes the four cardiac chambers are simultaneously in diastole [1]. ITBV represents the thoracic vascular distributional volume of a dye indicator injected in to a central vein [3]. GEDV and ITBV are closely related [2,7,8]. As GEDV can be determined more easily using cold saline [2], ITBV is estimated from GEDV in clinical routine. For clinical use and to compare individual patients, GEDV and ITBV are indexed to body surface area, yielding GEDV index (GEDVI) and ITBV index (ITBVI). Lower values of GEDVI or ITBVI are more frequently detected in volume-depleted patients [1]. These patients are likely to respond with an increase in cardiac index (CI) to a volume challenge. This is accompanied by an increase in GEDVI or ITBVI, whereas changes of CI induced by application of inotropic drugs leave GEDVI or ITBVI unchanged [1].

Further clinical validation of GEDVI was performed using transesophageal echocardiography [9-13]. Compared with continuous end-diastolic volume index, as well as left and right heart end-diastolic volume indices derived by modified pulmonary artery catheters, changes in GEDVI gave a better reflection of changes in cardiac preload in response to a volume challenge. Numeric values of GEDVI and echocardiographic volume indices show only a moderate correlation [9,10], explained in part by different techniques used for echocardiographic volume calculation [14].

Despite the usefulness of GEDV and ITBV for assessment of hemodynamic status, no validation study for the numeric values of these parameters has been carried out so far. Reference ranges for their indexed values were proposed by expert opinion to be 680 to 800 ml/m^2 ^for GEDVI and 850 to 1000 ml/m^2 ^for ITBVI. In a retrospective study, we found a considerable number of patients deviating from these proposed normal ranges, although clinically appearing adequately volume resuscitated [15]. The aim of the current study was to investigate the hypothesis that GEDVI acquired by transpulmonary thermodilution depends on age and gender in awake and spontaneously breathing subjects.

## Materials and methods

The study was approved by the Ethics Committee of the Bayerische Landesärztekammer, Munich, Germany. Informed consent was obtained from all patients.

### Study population

We included patients requiring elective brain tumor surgery at the Department of Neurosurgery, Klinikum Bogenhausen, a 1000-bed teaching hospital of the Technische Universität München, Germany. For perioperative monitoring and maintenance of anesthesia, patients undergoing craniotomy are routinely equipped with a central venous and an arterial line as standard of care in our department. Instead of the regular arterial line, a five french thermodilution catheter (PULSION PVPK2015L20-46N, PULSION Medical Systems AG, Munich, Germany) was placed in a femoral artery at induction of anesthesia and connected to a PiCCOplus thermodilution monitor (Version 7.0; PULSION Medical Systems AG, Munich, Germany).

Patients had to be at least 18 years old and to give informed consent to be included in the study. Exclusion criteria were inability or unwillingness to participate, missing or withdrawn informed consent, chronic atrial fibrillation, and known heart failure or pulmonary disease with dyspnea requiring supplemental oxygen. At study inclusion, the patient's body height and weight were measured.

### Thermodilution principle

After injection of a bolus of ice-cold saline through the central venous line into the right atrium, CO is computed from the area under the thermodilution curve obtained by a thermistor at the tip of the arterial catheter [16]. Temporal analysis of the thermodilution curve allows calculation of the central blood volumes [17]. The mean transit time (MTt) is the mean time from the start of injection to detection of the indicator by the arterial sensor, adjusted for recirculation [17]. The downslope time (DSt) describes the exponential decay of the thermodilution curve after its maximum [17]. Multiplication of the MTt with CO equals the total volume marked by the thermal indicator, the intrathoracic thermal volume (ITTV) [17]. Multiplication of the DSt with CO represents the largest compartment of the sequential mixing chambers of the thermal indicator, the pulmonary thermal volume (PTV) [17]. The difference between ITTV and PTV equals the GEDV [1]. ITBV is extrapolated by multiplying GEDV by a fixed factor of 1.25, offering acceptable accuracy in the clinical setting [18]. The difference between the ITTV and the ITBV equals the extravascular lung water (EVLW) [2].

### Data acquisition and processing

All monitoring data was stored using the PiCCOWin software (Version 7.0, PULSION Medical Systems AG, Munich, Germany). CO was indexed with body surface area (BSA) calculated from actual body weight and height. GEDV was indexed with BSA using measured height and predicted body weight (PBW), calculated differently for males and females: *PBW*_*male *_= 50 + 0.91 × (*height*_*cm *_- 152.4)and *PBW*_*female *_= 45.5 + 0.91 × (*height*_*cm *_- 152.4) [19]. BSA was determined by the Du Bois equation: *BSA *= 0.007184 × *length*_*cm*_^0.725 ^× *weight*_*kg*_^0.425 ^[20]. EVLW was indexed with PBW [21,22]. These calculations are performed automatically by the PiCCOplus device and not amenable for end user adjustment. From the monitor raw data, MTt and DSt were extracted.

### Study protocol

Preoperatively, patients were fasted overnight. Induction and maintenance of anesthesia, surgery and postoperative surveillance on the neurosurgical intensive care unit were performed as per the standards for our department and independent from the study. Thermodilution measurements were performed at least triplicate with 20 ml of iced saline and the mean of a series was taken. The current analysis considers the measurements performed in the morning before discharge of the patients from the ICU on the first postoperative day. Patients not ready for discharge on postoperative day one were excluded.

### Study size

Study size was planned using a bootstrapping strategy [23] on our previously analyzed retrospective data [15]. To achieve a power of above 85% for concurrent investigation of the dependencies of GEDV/GEDVI on age and sex, we aimed for analysis of at least 100 patients. This was reached after inclusion of 125 patients between July 2007 and June 2008.

### Statistical analysis

For statistical analysis we used the statistical environment R 2.8.1 [24].

The repeatability coefficient was defined as standard deviation divided by the mean of a measurement series.

Univariate analysis was performed using the Kruskal-Wallis-Test for CO, GEDV, GEDVI, MTt and DSt against predefined age groups in decades. The Welch t-test with correction for variance heterogeneity was used to compare the target parameters against gender and to screen for the impact of comorbidities and chronic medication. Gender differences in comorbidities and chronic medication were analyzed using the Chi square test or Fisher's exact test, as appropriate.

As GEDV and GEDVI showed no linear relationship with age, multivariate analysis was applied using generalized additive models [25] with GEDV and GEDVI as targets. Age was fitted with non-linear smoothing, sex as a factor, and height and actual body weight as linear explanatory variables. The combination of single parameters and their possible interactions were compared using significance values and the minimized Akaike Information Criterion [26].

## Results

**Figure 1:**
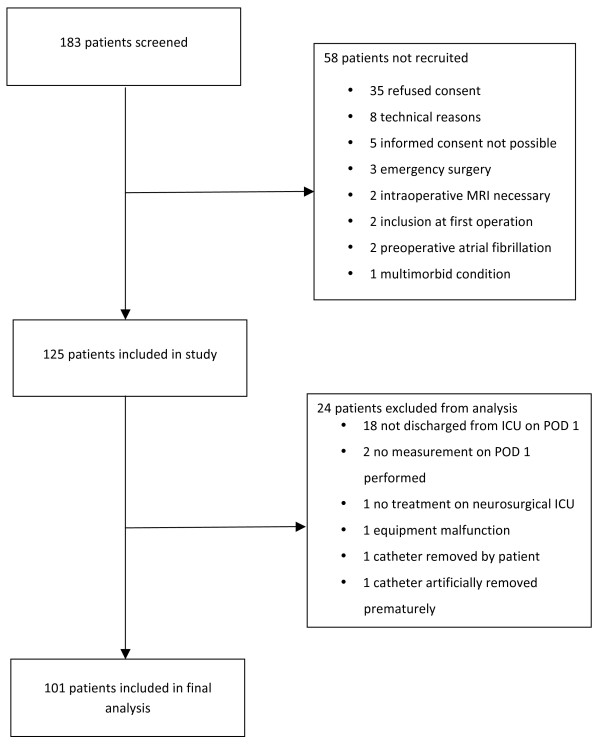
Flow of patient recruitment. ICU = intensive care unit; MRI = magnetic resonance imaging; POD = postoperative day.

On postoperative day 1, 101 patients were discharged and included in the study (Figure 1). Their demographic data, neurosurgical diagnosis and comorbidities, as well as preoperative medication are shown in Table 1. Age and body height were negatively correlated (r = -0.25, *P *= 0.011), while age and body weight showed a positive correlation (r = 0.18, *P *= 0.07).

**Table 1:** Preoperative comorbidities and demographic data

	all patients	male	female	
N	101	41	60	*P *value
Age [years] (range)	57 (21-83)	58 (24-79)	56 (21-83)	0.314
ASA score (range)	2 (1-3)	2 (1-3)	2 (1-3)	0.904
				
Height [cm] (range)	170 (151-194)	177 (163-194)	165 (151-182)	< 0.001
				
Weight [kg] (range)	78 (47-125)	84 (64-125)	73 (47-120)	< 0.001
BMI [kg/m^2^] (range)	26.83 (19.02-43.55)	27.09 (20.66-39.18)	26.66 (19.02-43.55)	0.131
BSA [m^2^] (range)	1.73 (1.37-2.19)	1.88 (1.64-2.19)	1.63 (1.37-1.93)	< 0.001
PBW [kg] (range)	63.2 (44.23-87.67)	72 (59.6-87.67)	57.2 (44.23-72.3)	0.195
**Tumor entities**				

Glioblastoma	15			
Astrocytoma	11			
Neurinoma	6			
Meningeoma	27			
Angioma	7			
Metastasis	16			
Pituitary	7			
Other	12			
**Preoperative comorbidities**				

Hypertension (%)	31 (30.7)	12 (29.3)	19 (31.8)	0.991
Myocardial infarction (%)	3 (3.0)	3 (7.3)	0	0.063
Stroke (%)	3 (3.0)	3 (7.3)	0	0.063
Adipositas (%)	11 (10.9)	4 (9.8)	7 (11.8)	1
Current smoker (%)	13 (12.9)	7 (17.1)	6 (10)	0.312
**Preoperative medication**				

ACE inhibitors (%)	19 (18.8)	8 (19.5)	11 (18.3)	0.927
β-blockers (%)	18 (17.8)	8 (19.5)	10 (16.7)	0.904
Proton pump inhibitors (%)	28 (27.7)	11 (26.8)	17 (28.3)	0.932
Anticonvulsives (%)	19 (18.8)	6 (14.6)	13 (21.7)	0.541
Diuretics (%)	14 (13.8)	6 (14.6)	8 (13.3)	0.927
Calcium antagonist (%)	7 (7.0)	5 (12.2)	2 (3.3)	0.115
Steroids (%)	37 (36.6)	18 (43.9)	19 (31.7)	0.280
Statins (%)	5 (5.0)	3 (7.3)	2 (3.3)	0.391

ACE = angiotensin converting enzyme; ASA = American Society of Anesthesiologists; BMI = body mass index; BSA = body surface area; n = number of patients; PBW = predicted body weight.

The median repeatability coefficient of all thermodilution series for CO was 6.0% (interquartile range (IQR) = 3.9% to 9.4%), for GEDV 7.4% (IQR = 5.4% to 10.5%), for MTt 4.0% (IQR = 2.5% to 6.1%) and for DSt 7.1% (IQR = 4.2% to 11.1%).

**Figure 2:**
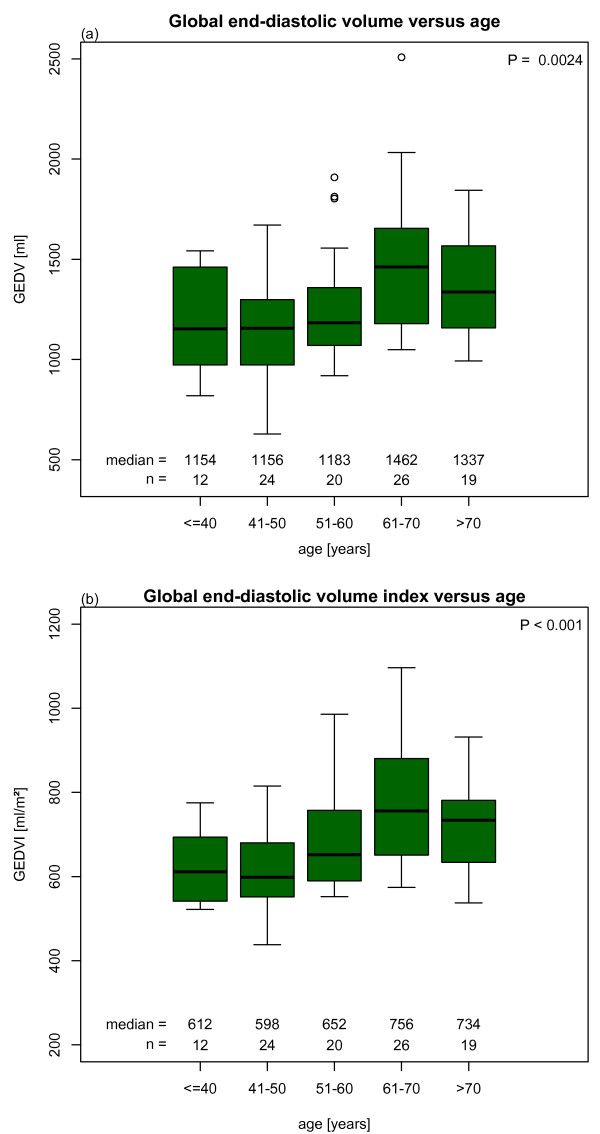
**(a) Global end-diastolic volume (GEDV) and (b) global end-diastolic volume index (GEDVI) versus age in predefined groups (univariate comparison)**.

**Figure 3:**
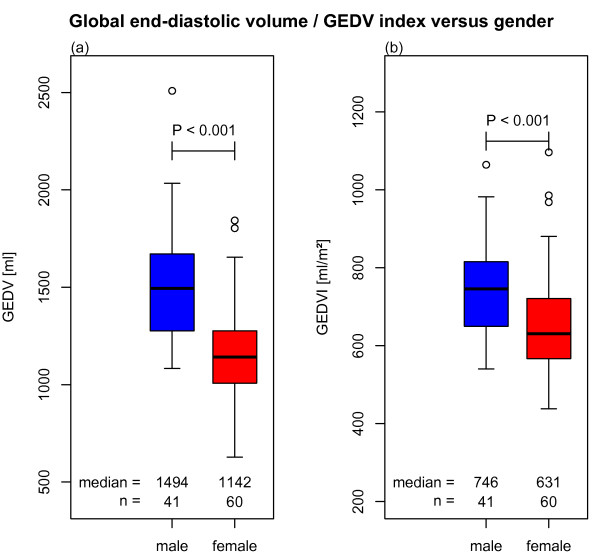
(a) Global end-diastolic volume (GEDV) and (b) global end-diastolic volume index (GEDVI) versus gender (univariate comparison).

Univariate analysis of GEDV and GEDVI showed significant differences between age groups (Figures 2a, b). The mean GEDV and GEDVI were significantly different between genders (Figures 3a, b).

The parameters CO, MTt and DSt are determinants of GEDV and their relationship with age and gender was examined. MTt was significantly different between age groups, with increasing values in higher decades (*P *= 0.0029). CO and DSt showed no significant difference between age groups (*P *= 0.36 and *P *= 0.067, respectively). CO and MTt were significantly higher in male patients (*P *= 0.004 and *P *= 0.05, respectively, Table 2). DSt showed no significant difference between genders (*P *= 0.3, Table 2).

**Table 2:** Hemodynamic parameters at discharge from the ICU

	all patients		male		female		
	mean (SD)	n	mean (SD)	n	mean (SD)	n	*P *value
**APsys [mmHg]**	139 (20)	98	141 (20)	39	138 (21)	59	0.559
**APmean [mmHg]**	94 (15)	98	96 (16)	39	93 (14)	59	0.324
**APdia [mmHg]**	66 (13)	98	68 (14)	39	65 (13)	59	0.141

**HR [1/min]**	77 (13)	98	74 (14)	39	79 (13)	59	0.074
**CO [l/min]**	7.16 (1.38)	101	7.55 (1.51)	41	6.78 (1.2)	60	0.004
**CI [l/min/m^2^]**	3.81 (0.63)	101	3.93 (0.6)	41	3.82 (0.6)	60	0.890

**MTt [seconds]**	18.5 (0.91)	101	19.5 (0.94)	41	17.9 (0.89)	60	0.05
**DSt [seconds]**	7.37 (0.63)	101	7.38 (0.63)	41	7.36 (0.71)	60	0.3
**GEDV [ml]**	1307 (310)	101	1509 (296)	41	1167 (235)	60	< 0.001
**GEDVI [ml/m^2^]**	693 (137)	101	750 (130)	41	653 (128)	60	< 0.001
**ITBV [ml]**	1634 (338)	101	1887 (370)	41	1459 (239)	60	< 0.001
**ITBVI [ml/m^2^]**	866 (171)	101	938(162)	41	816 (160)	60	< 0.001
**EVLW [ml]**	526 (164)	101	533 (97)	41	526 (200)	60	0.80
**EVLWI [ml/kg]**	8.5 (3.0)	101	7.4 (1.2)	41	9.3 (3.6)	60	< 0.001

SVR [dyn*s*cm^-5^]	1072 (517)	98	1035 (485)	39	1097 (539)	59	0.317
SVRI [dyn*s*cm^-5^m^-2^]	2001 (921)	98	2054 (829)	39	1965 (983)	59	0.374
**fluid balance [ml]**	1743 (1431)	100	1664 (1442)	41	1798 (1447)	59	0.911

Apdia = diastolic arterial pressure; APmean = mean arterial pressure; APsys = systolic arterial pressure; CI = cardiac index; CO = cardiac output; DSt = downslope time; EVLW = extravascular lung water; EVLWI = extravascular lung water index; GEDV = global end-diastolic volume; GEDVI = global end-diastolic volume index; HR = heart rate; ICU = intensive care unit; ITBV = intrathoracic volume; ITBVI = intrathoracic volume index; MTt = mean transit time; n = number of patients; SD = standard deviation; SVR = systemic vessel resistance; SVRI = systemic vessel resistance index.

The EVLW is a further derivative of CO, MTt and DSt. In contrast to GEDV, EVLW showed no significant difference between age groups and gender (*P *= 0.24 and *P *= 0.81, respectively, Table 2). Indexed to PBW, EVLWI was significantly higher in females (*P *< 0.001, Table 2), but not significantly different between age groups (*P *= 0.13).

Table 3 lists mean GEDV and GEDVI according to comorbidities and chronic medication. A significant difference was found for patients treated with statins. These patients were considerably older than the whole collective (71.8 years vs. 56.9 years, *P *< 0.001). As statin medication concerned five patients only, further analysis on subgroups or splitting on gender did not seem appropriate.

**Table 3:** GEDV and GEDVI according to comorbidities and preoperative medication

	GEDV [ml]			GEDVI [ml/m^2^]		
Comorbidities	yes	no	*P *value	yes	no	*P *value
	mean (SD)	mean (SD)		mean (SD)	mean (SD)	
**Hypertension**	1384 (288)	1276 (317)	0.100	717 (140)	684 (135)	0.281
**Myocardial infarction**	1660 (172)	1299 (309)	0.056	810 (69)	691 (69)	0.082
**Stroke**	1754 (224)	1296 (304)	0.064	866 (101)	689 (13)	0.086
**Adipositas**	1378 (261)	1301 (317)	0.390	650 (87)	700 (141)	0.115
**Smoker**	1252 (255)	1313 (338)	0.467	659 (91)	697 (146)	0.230
**Medication**						

**ACE-inhibitors**	1441 (304)	1279 (313)	0.051	719 (144)	689 (135)	0.417
**β-blockers**	1389 (256)	1292 (321)	0.179	725 (140)	687 (136)	0.310
**Proton pump inhibitors**	1240 (239)	1337 (333)	0.111	665 (100)	706 (148)	0.117
**Anticonvulsives**	1223 (263)	1331 (319)	0.134	666 (128)	701 (139)	0.299
**Diuretics**	1426 (332)	1291 (305)	0.171	690 (133)	722 (158)	0.475
**Ca2+antagonists**	1571 (338)	1290 (302)	0.072	788 (168)	688 (133)	0.210
**Steroids**	1375 (378)	1271 (259)	0.148	723 (163)	677 (116)	0.137
**Statins**	1619 (305)	1293 (278)	0.057	849 (124)	686 (133)	0.041

ACE = angiotensin converting enzyme; GEDV = global end-diastolic volume; GEDVI = global end-diastolic volume index; SD = standard deviation.

**Figure 4:**
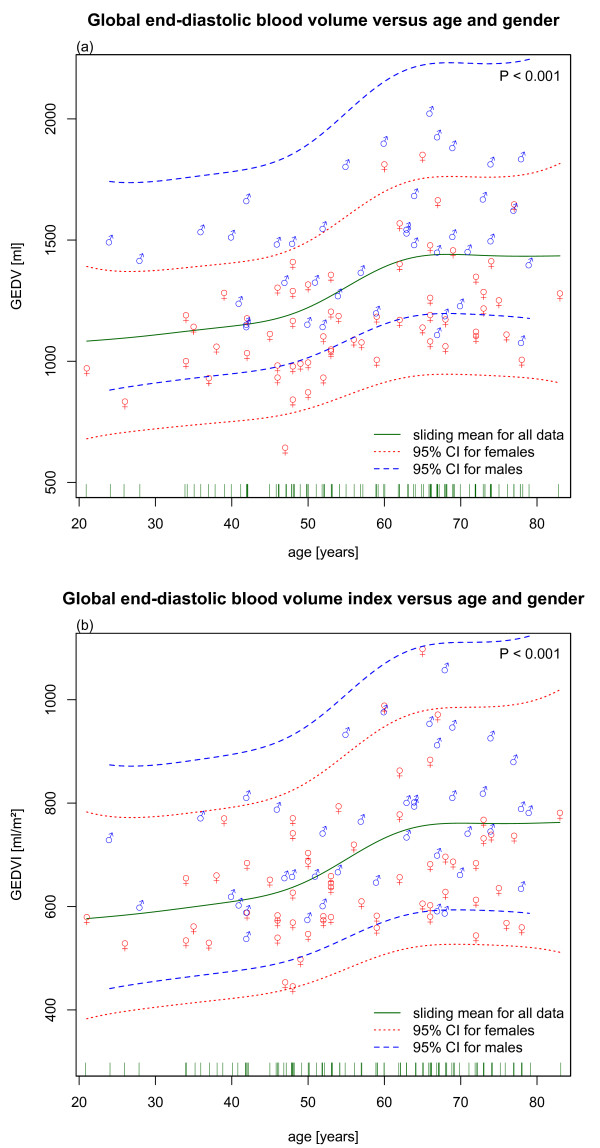
(a) Global end-diastolic volume (GEDV) and (b) global end-diastolic volume index (GEDVI) versus age using a generalized additive model. The continuous line represents the highly significant non-linear relationship for all data (*P *< 0.0001). The dotted and dashed lines show the 95% confidence interval (CI) for females and males. Single data points are shown with male and female symbols.

In multivariate modeling, the relationship of GEDV and GEDVI with age was highly significant and non-linear (Figures 4a, b). Male patients showed a mean GEDV of 131.1 ml more than females (95% confidence interval = 16.1 ml to 256.2 ml, *P *= 0.027). On average, each cm in body height accounted for an increase of 13.0 ml of GEDV (95% confidence interval = 6.2 ml to 19.8 ml, *P *< 0.001). Each kg actual body weight increased GEDV by 2.9 ml (95% confidence interval = 0.14 ml to 5.72 ml, *P *= 0.043).

After indexing GEDV to PBW, significant relationships for gender, size and weight persisted. On average, male sex accounted for a GEDVI increase of 67.3 ml/m^2 ^(95% confidence interval = 5.5 ml/m^2 ^to 134.5 ml/m^2^, *P *= 0.035). GEDVI increased by 3.7 ml/m^2 ^per cm height (95% confidence interval = 0.09 ml/m^2 ^to 7.38 ml/m^2^, *P *= 0.047). Weight was negatively correlated with GEDVI (-2.0 ml/m^2 ^per kg, 95% confidence interval = -3.50 ml/m^2 ^to -0.50 ml/m^2^, *P *= 0.010).

Adding interactions between coefficients as well as non-linear smoothing for height and weight did not improve the prediction models for GEDV and GEDVI. Adding statin medication as cofactor, which was suggested from the univariate results, showed no significant effect in multivariate analysis (*P *= 0.13 and *P *= 0.15, respectively).

Table 4 lists mean ranges for GEDVI values calculated with the final multivariate model according to the age groups defined for univariate analysis. As expected from Figure 4, the confidence intervals overlap considerably between groups and show a monotonous increase in mean value and width for higher age in both sexes.

**Table 4:** GEDVI means with 95% confidence intervals for males and females according to age groups

	GEDVI [ml/m^2^]	
**Age [years]**	**mean male (95% CI)**	**mean female (95% CI)**

**<= 40**	633 (456-880)	559 (402-779)

**41-50**	667 (485-916)	592 (432-812)

**51-60**	736 (536-1011)	654 (478-897)

**61-70**	802 (585-1101)	713 (520-977)

**>70**	812 (590-1117)	720 (520-997)

CI = confidence interval; GEDVI = global end-diastolic volume index

Univariate examination suggested a possible gender difference for EVLWI. Therefore, we performed an additional multivariate exploration with the predictors significant for GEDVI. Using stepwise deletion of the least important factor, the only parameter remaining significantly correlated with EVLWI was height (-0.11 ml/kg per cm, *P *= 0.001), while weight, gender and age showed no significant relationship (*P *= 0.65, *P *= 0.40, *P *= 0.10, respectively, in sequence of deletion).

## Discussion

The main finding of the current study is the dependence of the preload values GEDV and GEDVI on age and gender. Furthermore, our results show a large inter-individual variance, reflected in wide confidence intervals for the age-dependent means. The previously known and rather narrow normal ranges for GEDVI were defined on expert opinion only. As ITBV is calculated using GEDV and a fixed transformation factor, our findings also apply to ITBV and ITBVI estimated by single transpulmonary thermodilution.

The patients included in our study were without known hemodynamically relevant cardiopulmonary pathology in their medical history. For this reason, we did not perform routine echocardiography or a stress electrocardiogram for study inclusion. Admission to intensive care unit (ICU) was performed for postoperative safety reasons and not due to hemodynamic instability. No patient required vasoactive drugs or inotropic support when the thermodilution measurements were performed. All patients were breathing spontaneously and were discharged from the ICU shortly afterwards. We believe that our cohort resembles a representative normal cross-section of adults. Consequently, our data presents the first clinical series of values for the preload volumes GEDV and GEDVI in this population.

### Physiologic rationale

Analysis of the time parameters MTt and DSt from the transpulmonary thermodilution raw data revealed that MTt increases with age, while DSt shows no significant difference. Therefore, the ITTV derived from MTt increases with age and is higher in males. In contrast, the PTV derived from the DSt is independent of age and gender.

The difference between the thermal volumes ITTV and PTV equals the GEDV. Despite its name, the GEDV also includes the volume of the aorta from the aortic valve to the tip of the arterial thermistor [27]. The femoral catheter used in our study, and in most other investigations on transpulmonary thermodilution, has a length of 20 cm. In an adult it is placed with its tip roughly at the iliac bifurcation. It is well known that the aortic diameter increases with age and is larger in males than females [28-32]. Mao and colleagues studied 1442 consecutive asymptomatic subjects scheduled for coronary computed tomography (CT) angiography [28]. Measured with aortic contrast CT, the upper normal limits of the diameter of the ascending aorta were 35.6, 38.3 and 40 mm for females and 37.8, 40.5 and 42.6 mm for males in age groups 20 to 40, 41 to 60 and older than 60 years, respectively. Using an estimated aortic length of 50 cm, a 5 mm increase in luminal diameter would result in approximately 150 ml additional volume. This increase would explain the major part of our findings but does not take into account aortic elongation in elderly subjects [33]. The consequence, again, would be increased distribution volume of the thermal indicator [34].

End-systolic and end-diastolic volumes, measured with cardiac magnetic resonance tomography, are higher in male compared with female patients [35-40]. We also found comparable gender differences in GEDV in our study. Seemingly in contrast to our results, a decrease in cardiac volumes with age is described [35,37-39]. As we did not perform cardiac imaging in our patients, we are unable to further explain these findings. It is, however, conceivable that the increase in aortic diameter and length may offset the decrease in cardiac volume in older patients.

### Indexing problems

Indexing of hemodynamic variables is performed to remove differences between subjects for gender, weight and height. Therefore, theoretically, no significant contribution of any of these factors should persist. However, the contrary is found in our data and the literature [35-41]. Using BSA calculated with PBW for indexing of GEDV, yielding GEDVI, the influence of gender, height and weight remained significant confounders. PBW is dependent on gender and body height [19], but not on actual body weight. The negative correlation of GEDVI with weight in our multivariate analysis suggests that the indexing method overcorrects for heavier subjects.

Likewise, indexing EVLW using gender-specific PBW explains at least part of the higher female EVLWI values in our data. If indexing would be performed equally for both sexes instead of using a gender-specific formula, the difference in EVLWI, but not in GEDVI, would diminish (data not shown). Multivariate analysis with the predictors significant for GEDVI shows that height is negatively correlated with EVLWI, while gender, age and weight have no significant relationships. Therefore, we do not think there is sufficient evidence for a true EVLWI gender difference. The finding in univariate analysis is likely to be related to indexing of EVLW with PBW, which seems overly corrective for larger - more likely to be male - subjects.

### Clinical implications

The results of the current study imply that the use of the fixed normal ranges for targeting volumetric therapy is misleading. Although younger patients and females might get severely overhydrated aiming for the proposed normal ranges, older patients may erroneously be deprived of necessary volume. Clinical trials on preload optimization show a lack of consistency on hemodynamic goals and large heterogeneity in treatment effects [42]. Our results may explain part of these findings.

The wide confidence bands for GEDV and GEDVI in our data raise concern of targeting volume resuscitation with absolute values. Relative changes after volume expansion may better indicate volume status [27] and, in our opinion, require further study. If GEDVI changes substantially after a volume challenge, the patient is likely to be volume responsive. In contrast, if the EVLWI shows a pronounced increase, while GEDVI rises only marginally, this may be an indicator of overhydration.

### Limitations of the study

The patients in our study appeared to have normal cardiopulmonary function and were evaluated shortly before discharge from the ICU. The overnight fasting required preoperatively is described to have no impact on intravascular blood volume [43]. We cannot exclude a postoperative stress response that may influence cardiac performance or circulating blood volumes. However, a stress response may be present in volunteers or patients examined before induction of anesthesia. Conversely, any premedication may blunt a normal stress level.

Obviously, the patients included in our study were not healthy volunteers. They required craniotomy for removal of a brain tumor. However, we are unaware of any data indicating that patients requiring elective craniotomy present with an abnormal hemodynamic profile. As some of the patients had metastatic brain tumors, we cannot exclude that some of them may have been compromised by their underlying disease. No patient had undergone lobar lung resection, pneumonectomy or chemotherapy at the time of study inclusion.

Our study was not powered to detect a potential impact of chronic medication on preload volumes. However, according to our univariate analysis, the magnitude seems to be far lower than the relationship of preload volumes with age and gender found. In view of the large interindividual variance between subjects, any hypothetical confirmatory trial would have to include at least a ten-fold greater number of patients.

Finally, we did not investigate the influence of interventions such as a volume challenge or passive leg raising on the static preload parameter GEDV. Pulse pressure variation or stroke volume variation are dynamic indicators of cardiac preload and provide valuable information on the volume responsiveness of a patient [44]. Nevertheless, we do think that our findings may help to increase the physiologic understanding of volumetric preload parameters acquired by transpulmonary thermodilution.

## Conclusions

We provide evidence that the volumetric parameters GEDV and ITBV as well as their indexed versions GEDVI and ITBVI are dependent on age and gender in spontaneously breathing patients without hemodynamic support and show wide confidence intervals due to a large variance between individuals. Targeting resuscitation using fixed ranges of preload volumes acquired by transpulmonary thermodilution without concern for the individual patient's age and gender seems not to be appropriate. Future studies investigating whether these findings translate into optimized volume therapy in acutely ill patients are clearly warranted.

## Key messages

• The preload volumes GEDV and ITBV are dependent on age and gender.

• The age and sex dependence of GEDV and ITBV is persistent after indexing to BSA.

• GEDVI and ITBVI show wide confidence intervals in spontaneously breathing patients due to a large variance between individuals.

• Targeting resuscitation using fixed ranges of GEDVI or ITBVI without concern for age and gender is not appropriate.

## Abbreviations

BSA: body surface area; CI: cardiac index; CO: cardiac output; CT: computed tomography; DSt: downslope time; EVLW: extravascular lung water; GEDV: global end-diastolic volume; GEDVI: global end-diastolic volume index; ITBV: intrathoracic blood volume; ITBVI: intrathoracic blood volume index; ITTV: intrathoracic thermal volume; MTt: mean transit time; PBW: predicted body weight; PTV: pulmonary thermal volume.

## Competing interests

The study was supported by PULSION Medical Systems AG, Munich, Germany, who provided thermodilution catheters and additional unrestricted funding. The sponsor was not involved in study planning, acquisition, analysis or presentation of the data or the preparation of the manuscript. All authors declare that they have no competing interests.

## Authors' contributions

All authors had full access to all of the data in the study and contributed intellectual content to the final form of the manuscript. SW wrote the study protocol, obtained funding, collected and analyzed data and wrote the manuscript. AR collected and analyzed data, checked the data integrity and contributed to the manuscript. JL had the idea of the study and collected and analyzed data. CBL reviewed the study protocol and provided important intellectual content. PF analyzed data, contributed and edited the revisions of the manuscript. LS collected and analyzed data and contributed and edited all revisions of the manuscript.
